# Genome-wide analysis of the *WOX* gene family and function exploration of *RhWOX331* in rose (*R*. ‘The Fairy’)

**DOI:** 10.3389/fpls.2024.1461322

**Published:** 2024-09-03

**Authors:** Lian Duan, Zhihui Hou, Wuhua Zhang, Shuang Liang, Minge Huangfu, Jinzhu Zhang, Tao Yang, Jie Dong, Daidi Che

**Affiliations:** ^1^ College of Horticulture and Landscape Architecture, Northeast Agricultural University, Harbin, China; ^2^ Key Laboratory of Cold Region Landscape Plants and Applications, Harbin, China

**Keywords:** *Rosa hybrida*, *WOX* gene family, *RhWOX331*, adventitious roots, gene function analysis

## Abstract

*WOXs* are a class of plant-specific transcription factors that play key roles in plant growth and stress responses. However, the mechanism by which *WOXs* influence adventitious root development in *Rosa hybrida* remains unclear. In this study, *RcWOX* gene family in rose was identified and phylogenetically analyzed using bioinformatics analysis. A total of 381 *RcWOX* gene members were localized on seven chromosomes except of nine members. The main *cis*-acting elements involved in hormonal, light, developmental, and abiotic stress responses were identified in the promoters of *RcWOX* genes, suggesting their regulation by these signals. Nine *RhWOX* genes had significant different expression during rooting process of rose. *RhWOX331*, *RhWOX308*, *RhWOX318* were positive with the formation of rose roots. RhWOX331 was positively involved in the formation of adventitious root primordia, which gene coding a transcription factor localized in the nucleus. The HOX conserved domain in the protein contributed to the self-activating activity of RhWOX331. We obtained genetically modified Arabidopsis to validate the function of *RhWOX331*. Overexpression of *RhWOX331* gene alleviated the inhibition of root length of *A. thaliana* primary roots by high concentration of IBA and NPA, and significantly increased the number of lateral roots on the primary roots, as well as the height of *A. thaliana* plants. Additionally, *RhWOX331* promoted adventitious root formation in A. thaliana and mitigated hormonal inhibition by exogenous 6-BA, NPA, and GA_3_. The *RhWOX331* promoter contained cis-acting elements such as ABRE, Box 4 and CGTCA-motif et.al. GUS activity analysis showed that the gene acted at the cotyledon attachment site. Taken together, these studies identified a significant expansion of the *RcWOX* gene family, inferred roles of certain branch members in adventitious root formation, elucidated the function of *RhWOX331* in adventitious root initiation, and laid the foundation for further research on the function of *WOX* gene family in roses.

## 1 Introduction

In agriculture, forestry and horticulture, plant organ regeneration was often utilized in cuttings propagation practices to obtain a large number of plants that retained the parent’s good traits (De Klerk et al., 1999) For woody plants, The incidence of adventitious roots (ARs) during the propagation of cuttings determined the survival and efficiency of propagation of the species. ARs can be initiated from column sheath cells in the hypocotyl, thin-walled cells in the phloem or xylem, young secondary phloem cells, or cells of the inter bundle formation layer close to phloem cells (Bellini et al., 2014). The formation of adventitious roots was regulated by a combination of external environment, endogenous substances, and other factors, including light, water, spike age, and phytohormones (Bannoud and Bellini, 2021).

The WUSCHEL (WUS) homeobox transcription factor was a plant-specific transcription factor with a conserved “helix-loop-helix-turn-helix” motif comprising 60-66 amino acid residues (van der Graaff et al., 2009). During the phylogenetic process of higher plants, the *WOX* genes had evolved into three major classical clades: the modern/WUS clade, the ancient clade, and the intermediate clade. The modern evolutionary clade included *WUS*, *WOX1~7*, totaling 8 members, and the intermediate clade included 4 members, *WOX8*, *WOX9*, *WOX11* and *WOX12*. The ancient clade members contained three genes, *WOX10*, *WOX13* and *WOX14* (Liu and Xu, 2018). Studies of the *WOX* gene family in *Arabidopsis thaliana* (Ohmori et al., 2013), *Populus trichocarpa* (Shuang et al., 2019), and *Picea abies* (Palovaara et al., 2010) had revealed that members of *WOX* gene family in each clade interacted with hormones to regulate plant growth and development processes. The *WOX* gene family played crucial regulatory roles during key stages of plant development such as embryo formation, stem cell maintenance, and organogenesis (Tanaka et al., 2015; Zhang et al., 2017), which were mediated by promoting cell division or inhibiting premature cell differentiation (Laux et al., 1996). These regulatory effects were likely achieved through interactions between *WOX* genes and hormones.

In modern clade, *AtWUS* regulated anther and ovule development (Reiser et al., 1995), and it also interacted with *CLAVATA3* (*CLV3*) to maintain the balance between proliferation and differentiation of stem tip meristems (Laux et al., 1996). *CsWUS* overexpression increased the number of sepals, petals and carpels in *Cucumis sativus* (Che et al., 2020). *AtWOX2* gene was expressed mainly in the apical cells of early embryonic development and regulated embryo formation (Liu and Xu, 2018). Overexpression of *AtWOX4* gene promoted radial growth of primary roots (Zhang et al., 2019). Among the genes in the intermediate clade, *AtWOX8* and *AtWOX9* were co-expressed in the pituitary cells of the embryo, promoted embryo development, and also functioned to maintain cell proliferation in the apical and root tip meristematic tissues (Liu and Xu, 2018). Overexpressing *PeWOX11a* or *PeWOX11b* in poplar not only enhanced adventitious root formation on the plugs, but also induced ectopic rooting in the aboveground part of transgenic poplar (Li et al., 2018). *OsWOX11* gene was expressed in the region of proliferative root tip cells and regulated the emergence and growth of crown roots in rice (Cheng et al., 2014). There were fewer members in the ancient clade, among which *AtWOX13* functioned in early stages of root development and in organs with high proliferation, *AtWOX14* gene expressed in *A. thaliana* primary roots, lateral root primordia, and floral organs, and inhibited cell differentiation (Deyhle et al., 2007). Expression of *SkWOX13B* in stone pine plants was closely related to root organogenesis (Ge et al., 2016).

As the premier among the world’s top four cut flowers, *Rosa hybrida* exhibits exceptionally high commercial value and possesses a unique cultural significance. In the genus *Rosa*, the ability to generate adventitious roots directly influences cutting survival and is a decisive factor in the garden application of *Rosa* species. *WOX* genes also regulated the growth and development of *Rosa* genus. The *RcaWOX1* gene from *Rosa canina* was induced by auxin and expressed at the early stage of healing tissue formation, overexpressing this gene increased the number of lateral roots and induced the up-regulated expression of *AtPIN1* and *AtPIN7* in *A. thaliana* (Gao et al., 2014). Overexpression of *RcWUS* induced the transformation of parenchyma cells in the root cortex into meristematic tissue cells, leading to the ectopic occurrence of adventitious shoots at the root tip (Jiang et al., 2012). The rooting ability of rose was influenced by factors such as genotype, lignification level of the cuttings, hormones, and environmental conditions. However, the molecular mechanisms underlying rose rooting remained unclear. This study provided a comprehensive overview of the *WOX* gene family in roses, investigating the expression patterns and functions of *RhWOX331* in adventitious rooting. It established a solid theoretical basis for further research on *RhWOX* genes involved in organogenesis in roses.

## 2 Materials and methods

### 2.1 Identification and phylogenetic analysis of *WOX* gene family members in rose

Genome and protein sequences of *Rosa chinensis*, *Rosa rugosa* and *Rosa multiflora* were obtained from the Rosaceae Genome Database (GDR) (Raymond et al., 2018; Jung et al., 2019). All *WOXs* in rose were identified using the Pfam protein family database (Mistry et al., 2021) by downloading the Hidden Markov Model (HMM) file for the WOX structural domain (PF00046) and setting a threshold of 1e^-5^. The core sequences of *RcWOXs* were verified using the SMART program and conserved domain database (CDD) (Wang et al., 2023). The protparam tool from the Expasy website (https://web.expasy.org/protparam/) was used to predict basic characteristics (amino acid length, amino acid composition, isoelectric point, etc.) of the obtained *WOX* family members (Walker, 2005). Each *RcWOX* gene family member was named according to their position on the chromosome using TBtools II (Chen et al., 2023). The sequences of *WOXs* in *A. thaliana* (Lamesch et al., 2012), *Nicotiana tabacum*, and *Populus trichocarpa* were downloaded from NCBI (https://www.ncbi.nlm.nih.gov/). MEGA11 (Tamura et al., 2021) was used to perform multiple sequence comparisons under default parameters, and a phylogenetic tree was constructed using the Neighbor-Joining method (bootstrap: 1000).

### 2.2 Analysis of *WOXs* of rose structure and conserved motifs

Multiple sequence comparisons were performed using ClustalW in MEGA11 under default parameters to further analyze the characteristic structural domains of RcWOX proteins and manually adjust the amino acid sequences. GSDS (Hu et al., 2015) was used for exon-intron structure visualization of *RcWOX* genes. RcWOXs protein motifs were analyzed using MEME (Bailey et al., 2009) under the parameter maximum motif number of 20.

### 2.3 Chromosomal localization, collinearity analysis and *cis*-acting element prediction of the *RcWOX*

Localization of all *RcWOX* genes to rose chromosomes based on physical location information using TBtools II (Chen et al., 2023). Utilizing TBtools II for collinearity analysis of the *WOX* gene family in rose with the WOX gene families of *A. thaliana* and *P. trichocarpa*. Promoter *cis*-acting regulatory elements were analyzed in the 2 Kb region upstream of the rose *WOXs* using PlantCARE (Lescot et al., 2002), and *WOX* gene family was visualized by TBtools II.

### 2.4 Plant materials and growth conditions

*R*. ‘The Fairy’ and *Nicotiana benthamiana* were grown in the Northeast Agricultural University (Harbin City, Heilongjiang Province, China) under a 16 h light/8 h dark at 25°C cycle. *A. thaliana* was grown under 14 h light/10 h dark conditions with a temperature range of 22-23°C and relative humidity between 40-60%.

### 2.5 Quantitative real-time PCR

Total RNA of plants leaves and roots was isolated with the FastPure Universal Plant Total RNA Isolation Kit (Vazyme Biotech Co., Ltd., Nanjing, China), and transcribed into cDNA using the HiScript III 1st Strand cDNA Synthesis Kit (+gDNA wiper) (Vazyme Biotech Co., Ltd., Nanjing, China). HiScript II QRT SuperMix for qPCR (Vazyme Biotech Co., Ltd., Nanjing, China) was used for qPCR. The determination of gene expression levels refers to previous research descriptions (Dong et al., 2021). The 2^−ΔΔCT^ quantification method (Schmittgen and Livak, 2008) was used to calculate the relative expression levels. *RhActin* (Fan et al., 2023) were selected as reference genes in *Rosa hybrida*. All experiments were conducted with three biological replicates, each containing three technical repeats. Define a total of 8 stages from US to CS7 based on the cutting time of cuttings. US: 0 d; CS1: 15 min; CS2: 1 d; CS3: 3 d; CS4: 5 d; CS5: 10 d; CS6: 15 d; CS7: 20 d. Primers used for RT-qPCR were listed in **Supplementary Table S2**.

### 2.6 Subcellular localization of RhWOX331

The full-length *RhWOX331* gene, lacking a stop codon, was inserted into *Kpn*I and *Bam*HI sites (Takara, Beijing, China) of the pGAMBIA1300-sGFP vector using the *pEASY* ^®^-Basic Seamless Cloning and Assembly Kit (TransGen Biotech, Beijing, China). The constructed vector pGAMBIA1300-*RhWOX331*-sGFP was transformed into *Agrobacterium tumefaciens* GV3101 (WeiDi Biotechnology, Shanghai, China), and subcellular localization was performed according to the previous research (Li et al., 2023). The infection solution (200 μM acetosyringone (AS), 10 mM 2-morpholinoethanesulphonic acid (MES), and 10 mmol/l MgCl_2_) containing either pGAMBIA1300-*RhWOX331*-sGFP or pGAMBIA1300-sGFP were injected into the subepidermal cells of 4-week-old *Nicotiana benthamiana* leaves. After 2 days of dark incubation at 23 °C, the subcellular localization of RhWOX331 was visualized and photographed using a laser-scanning confocal microscope (FV3000, Olympus, Japan) at 488 nm.

### 2.7 Yeast self-activation analysis of *RhWOX331*

The full-length *RhWOX331* gene was inserted into *Nde*I and *Eco*RI (Takara, Beijing, China) sites of the pGBKT7 vector using the *pEASY* ^®^-Basic Seamless Cloning and Assembly Kit (TransGen Biotech, Beijing, China). The *p*GADT7-T+*p*GBKT7-p53 (positive control), *p*GADT7-T+*p*GBKT7-lam (negative control), and *p*GBKT7-*WOX331-1*, *p*GBKT7-*WOX331-2*, *p*GBKT7-*WOX331-3* plasmids were transformed into Y2HGold yeast competent cells (WeiDi Biotechnology, Shanghai, China). After 2 days of cultivation at 28°C, yeast colonies were selected and cultured in SD/-Trp/-Leu liquid yeast medium at 28°C and 200 rpm. Centrifuge yeast at 4000 rpm for 1 minute to collect the yeast cells. The Y2HGold yeast containing the recombinant plasmid was resuspended in sterile water until its OD600 reached 0.2. The suspended culture was diluted to 1X, 10X, and 100X concentrations. The positive control and negative control diluted yeast solution was placed on SD/-Trp/-Leu/-His/-Ade/X-α-gal solid medium, the diluted yeast solution transforming *p*GBKT7-*WOX331-1*, *p*GBKT7-*WOX331-2*, *p*GBKT7-*WOX331-3* was placed on SD/-Trp/-His/X-α-gal solid medium and cultured at 28°C. After 36-48 h of incubation, the self-activating activity of *RhWOX331* was assessed based on the blue coloration of the yeast.

### 2.8 Genetic transformation and identification of transgenic *RhWOX331* in *A. thaliana*

pGAMBIA1300-*RhWOX331*-sGFP was transformed in *A. thaliana* with floral dip transformation method (Bent, 2006). Seeds of *A. thaliana* were collected and sown, until obtaining T3 generation plants. Transgenic *A. thaliana* were identified by PCR using WOX331F and WOX331R as primers (**Supplementary Table S2**). The seeds of transgenic *A. thaliana* were sterilized and sown in 1/2 MS medium (20 g/L sucrose + 8 g/L agar), and different hormones were added to the medium according to different treatments: CK: no hormone; IBA: 0.25 mg/L IBA; 6-BA: 0.5 mg/L 6-BA; GA_3_: 1 mg/L GA_3_; NPA: 10 μM NPA. The phenotypes of the primary roots of *A. thaliana* were determined after 14 days. At 14d, the main roots were removed and transferred to B5 medium (30 g/L sucrose + 8 g/L agar), and different hormones were added to the medium according to different treatments (hormone concentration as above), and the phenotypic changes of adventitious roots were observed.

### 2.9 Analysis of the GUS activity of *RhWOX331* promoter

The 2113 bp sequence upstream of the start codon of *RhWOX331* was divided into three segments. *p*WOX331 replaced 35S in PBI121 and construct *p*WOX331-1::*GUS*, *p*WOX331-2::*GUS* and *p*WOX331-3::*GUS* vectors with *Bam*HI and *Hin*dIII restriction site. Primers were listed in **Supplementary Table S2**. Transgenic *A. thaliana* overexpressing *p*WOX331-1::*GUS*, *p*WOX331-2::*GUS* and *p*WOX331-3::*GUS* were immersed in GUS staining solution (Coolaber, Beijing, China) and kept warm at 37°C for 1 h. Using 70% ethanol for decolorization 2~3 times, and the material was observed under the *in vitro* microscope (Olympus SZX2-ILLTQ). P1, P2, and P3 represent *A. thaliana* transformed with *p*WOX331-1:: *GUS*, *p*WOX331-2:: *GUS*, and *p*WOX331-3:: *GUS*, respectively. GUS activity was assessed using the previous method (Koo et al., 2007).

### 2.10 Statistical analyses

Statistical analyses were conducted with IBM SPSS v25.0 (SPSS Inc., Chicago, IL, USA). Least Significant Difference (LSD) test was performed in order to compare the statistical validity of data. Significance was set at p< 0.05. Three biological replicates were used for each assay. TBtoolsII software was used to create the conserved domains, motifs, gene structure. GraphPad Prism 8.0.0 (GraphPad Software San Diego, California USA) were used to plot graphs.

## 3 Results

### 3.1 Identification of *WOXs* in rose

The 381 members of the rose *WOX* gene family were finally identified in the whole rose genome, and they were named *RcWOX1*-*RcWOX381* based on their positions on the chromosome (**Supplementary Table S1**). The physicochemical properties of the 381 *WOX* genes revealed that the number of amino acids ranged from 81 to 400, and the theoretical isoelectric points ranged from 4.56 to 10.55, with 87.9% of them having isoelectric points lower than 7, indicating that they were mostly acidic proteins. The instability coefficients ranged from 36.55% to 86.47%, with 2.1% of the members having instability coefficients lower than 40%, and most of the WOX proteins were unstable proteins. The relative molecular mass of RcWOX335 was the largest at 44.66 KDa, and the relative molecular mass of RcWOX275 was the smallest at 9.87 KDa.

### 3.2 Phylogenetic analysis

**Figure 1:**
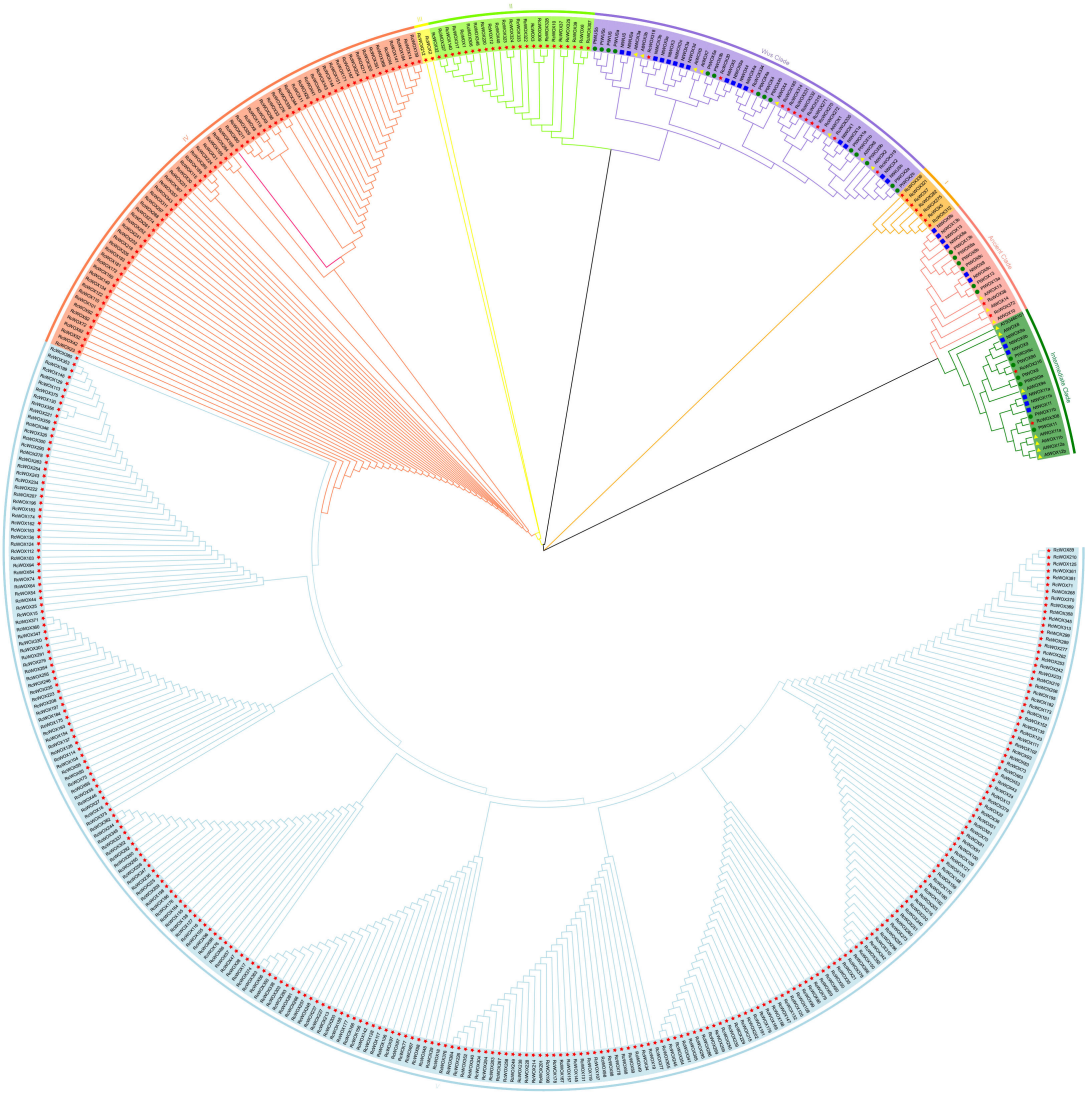
Phylogenetic analysis of the *RcWOX* gene family of *R. chinensis* and *N. tabacum*, *A. thaliana*, *P. trichocarpa*. Proteins from *R. chinensis*, *N. tabacum*, *A. thaliana* and *P. trichocarpa* were respectively denoted by the prefixes Rc, Nt, At, and Pt, respectively. They were divided into eight major phylogenetic clusters: ancient clade, intermediate clade, WUS clade, clade I, clade II, clade III;, clade IV, and clade V. Each clade was indicated by different colors. Bootstrap:1000.

In order to explore the phylogenetic relationships of *WOX*s in rose and other model plants, a phylogenetic tree was constructed based on the sequences of 453 WOX proteins from rose (381), *P. trichocarpa* (26), *N. tabacum* (28) and *A. thaliana* (18) (**Figure 1**). The phylogenetic tree analysis showed that the 453 genes were clearly divided into eight clades: ancient clade, intermediate clade, modern/WUS clade, clade I, clade II, clade III, clade IV, clade V. Among these, the *RcWOXs* in the classical clades including ancient, intermediate, and modern/WUS clades were more closely related to *N. tabacum*, *A. thaliana*, and *P. trichocarpa WOXs*. On the contrary, *WOX* genes in rose belonging to clades I to V had no homologous genes with *P. trichocarpa*, *N. tabacum* and *A. thaliana WOX* genes. Ancient clade contained 2 genes in rose, 3 genes in *A. thaliana*, 6 genes in *P. trichocarpa*, 6 genes in *N. tabacum*. Intermediate clade contained 2 genes in rose, 7 genes in *A. thaliana*, 6 genes in *P. trichocarpa*, 6 genes in *N. tabacum*. Modern/WUS clade contained 13 genes in rose, 8 genes in *A. thaliana*, 14 genes in *P. trichocarpa*, 16 genes in *N. tabacum*. Clades I to V contained 364 members, all of which originated from rose. Clade V was the largest clade, containing 265 members. The results show that there are a large number of similar redundant genes in rose *WOX* gene family, and they are distantly homologous to the *WOX* family members of the ancient, intermediate, and modern/WUS clades.

**Figure 2:**
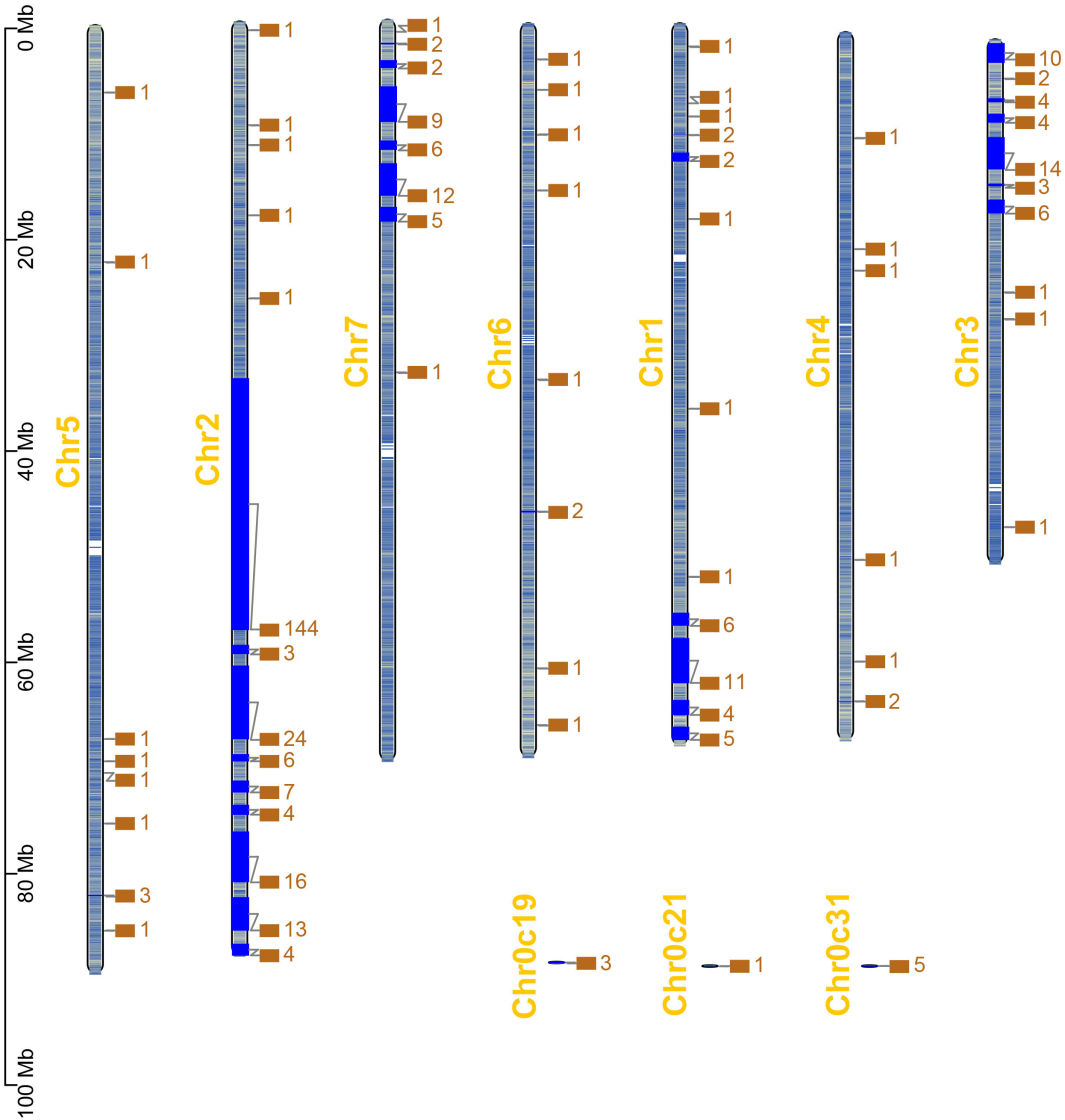
Chromosomal distribution of *RcWOX* genes in *R. chinensis*.

Based on the chromosomal location information of *RcWOXs* in *R. chinensis*, the positions of 381 *RcWOX* members on the chromosomes were visualized and analyzed (**Figure 2**). *RcWOXs* were distributed on all seven chromosomes, with a total of 226 *RcWOX* genes on chromosome 2, 46 *RcWOX* genes on chromosome 3, 38 *RcWOX* genes on chromosome 7, 36 *RcWOX* genes on chromosome 1, 10 *RcWOX* genes on chromosome 5, 9 *RcWOX* genes on chromosome 6, 7 *RcWOX* genes on chromosome 4, and 9 *RcWOX* genes not localized on any chromosome. *WOX* genes were most densely distributed on chromosome 2.

Conserved domain analysis of 381 *RcWOX* family members revealed the presence of two conserved domains: Homeodomain superfamily and Homeobox (**Supplementary Figure S1B**). In order to study the structure of RcWOXs, a figure depicting the RcWOX structure was created (**Supplementary Figure S1A**), which showed that motifs 10 and 15 were present in all members of *RcWOXs* of classical clades. In contrast, the vast majority of the members in clades I to V contained motifs 1, 2 and 4. The gene structure figure also indicated that 86.8% of members in clades I to V and 29.4% of members in classical clades lacked UTRs (**Supplementary Figure S1C**). Analysis of the amino acid sequences of clade V revealed that most of the proteins contained the amino acid domains SIMEQRGBYHQBIBTLPLFPMHGEDILGNMKTTSEGGGGGYGG and G/DSHISLELSLNSYRDADMA, corresponding to motifs 2 and 4. For clade IV of *WOXs* in rose, most proteins contained the amino acid domains HQEIETLMHGEDI and YGQIEDKNVFFWFQNLKA, which were absent in classical clades. These findings suggest significant differences in amino acid sequences, conserved domains, and intron distribution between *WOX* members of classical clades and clades I to V, implying potential functional distinctions.

The *cis*-acting elements within the upstream 2000bp of the initiation codon of 381 *WOX* genes in rose were involved in hormone, environment, growth and development (**Supplementary Figure S2**). Hormone-related *cis*-acting elements were salicylic acid-induced (W-box), jasmonic acid signaling pathway (MYC), and gibberellin response element (P-box). *Cis*-acting elements involved in environment including light-responsive (G-box, Sp1, TGACG-motif, TCCC-motif) and trauma response (WUN-motif). MYB, GCN4-motif, Circadian clock belonged to growth and development-related *cis*-acting elements. It was observed that *WOX* genes within the same clade of the phylogenetic tree exhibited similar *cis*-acting elements. These findings suggest that *WOX* genes in rose may be regulated by a diverse array of phytohormones, biotic and abiotic stimuli, influencing plant growth and development.

**Figure 3:**
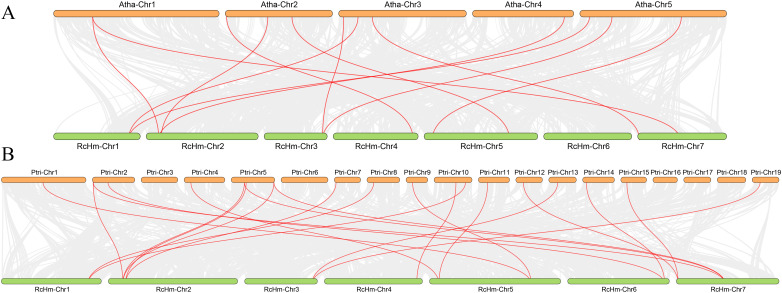
Collinearity analysis of the *WOXs* between *R. chinensis* both *A. thaliana* and *P. trichocarpa*. **(A)** Collinearity analysis of the *WOXs* between *R. chinensis* and *A. thaliana*. **(B)** Collinearity analysis of the *WOXs* between *R. chinensis* and *P. trichocarpa*.

In order to further investigate the interspecific evolutionary relationship of *WOXs*, intergenic collinearity analysis was performed between roses and model plants, such as *A. thaliana* (**Figure 3A**) and *P. trichocarpa* (**Figure 3B**). It was found that there were 12 homologous gene pairs between 381 *WOX* genes of rose and 15 *AtWOX* genes of *A. thaliana*, and 21 homologous gene pairs between 381 *WOX* gene family members of rose and 26 *PtWOX* genes of *P. trichocarpa.* These results suggest a high number of homologous gene pairs between rose *WOX* genes and both *AtWOXs* and *PtWOXs.*

### 3.3 Analysis of *RhWOXs* expression patterns

**Figure 4:**
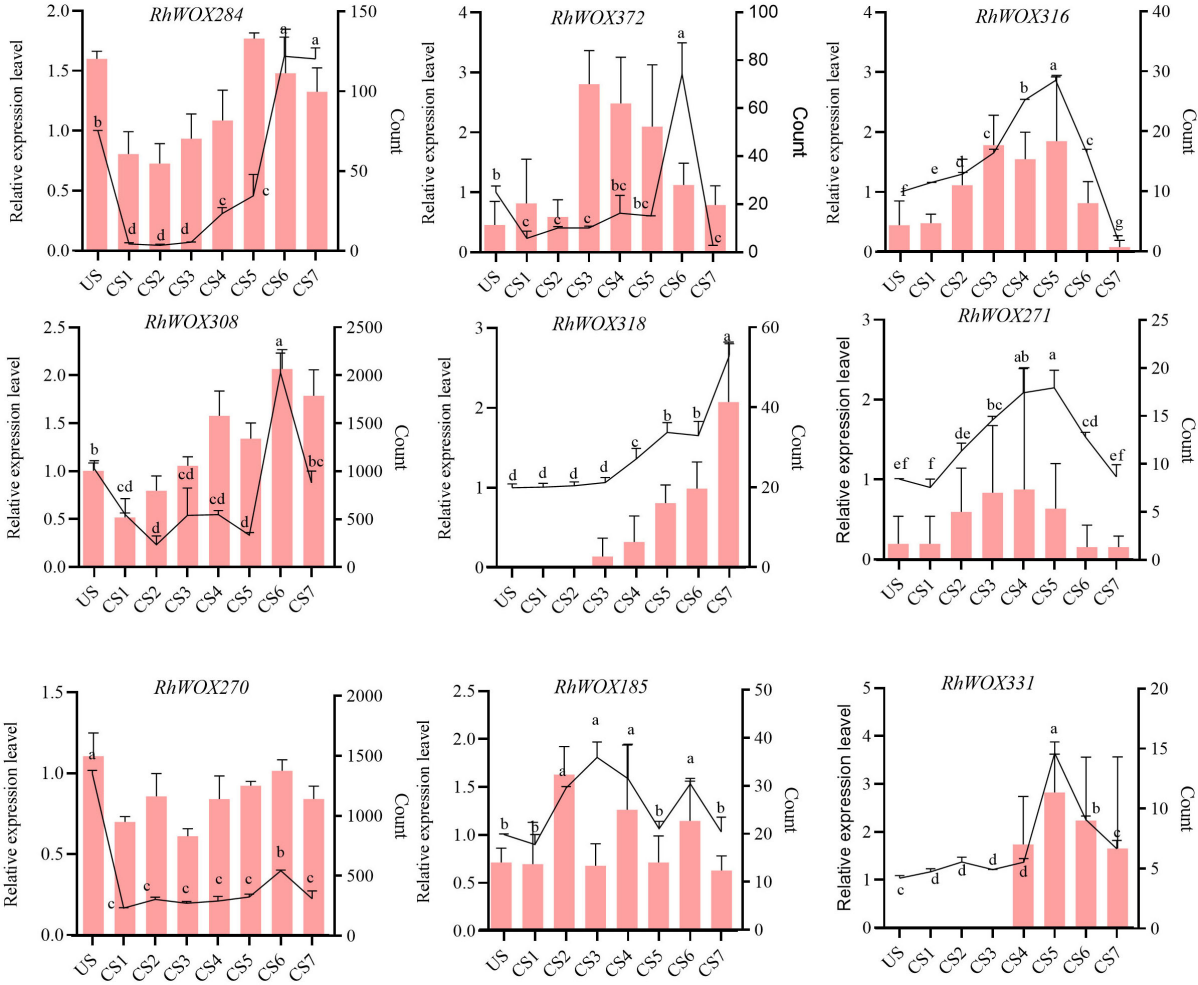
Relative expression of the *RhWOX* gene family during rooting at the stem nodes of *R*. ‘ The Fairy ‘ scions. The line graph shows the relative expression by RT-qPCR and the bar graph shows the gene count values determined by RNA-seq. The horizontal coordinates indicate the time of cuttings of *R*. ‘The Fairy’. US: 0 d; CS1: 15 min; CS2: 1 d; CS3: 3 d; CS4: 5 d; CS5: 10 d; CS6: 15 d; CS7: 20 d. Different lowercase letters indicate significant differences among different treatments.

The expression levels of *WOX* genes during adventitious root formation in roses indicated relatively higher expression levels in classical clades, with almost all members in clades I to IV showing no expression. Therefore, we selected *RhWOX* genes in ancient, intermediate, and modern/WUS clades. Combining the expression data of *WOXs* transcripts during adventitious rooting process of *R. hybrida*, nine *RhWOXs* were finally identified (**Figure 4**). Expression analysis of *RhWOXs* gene family members during rooting of single-node spikes of rose showed that *RhWOX284* was down-regulated 15 minutes after cutting, and up-regulated during leaf production. *RhWOX372*, *RhWOX316* and *RhWOX271* were up-regulated in the mid-root stage of CS3~CS5, and down-regulated in the root elongation stage. *RhWOX308* and *RhWOX270* were initially down-regulated after pruning, and these genes were significantly up-regulated as the stem cells divided and root primordia formed. *RhWOX318* gradually activated during root tip formation, exhibiting peak activity during root elongation. *RhWOX185* showed significant up-regulation during CS2 stage. *RhWOX331* remained low until root primordium formation (CS1~CS4), exhibited significant up-regulation during CS4~CS5, and then down-regulated during the period of root tip formation and root elongation, showing strong correlation with root primordium development. RT-qPCR data corroborated RNA sequencing results, with *RhWOX331* showing significant positive correlation with root primordium differentiation, Thus, we speculate that *RhWOX331* gene play a key role in the development of adventitious roots in *R. hybrida.*

### 3.4 Characterization of *RhWOX331*

**Figure 5:**
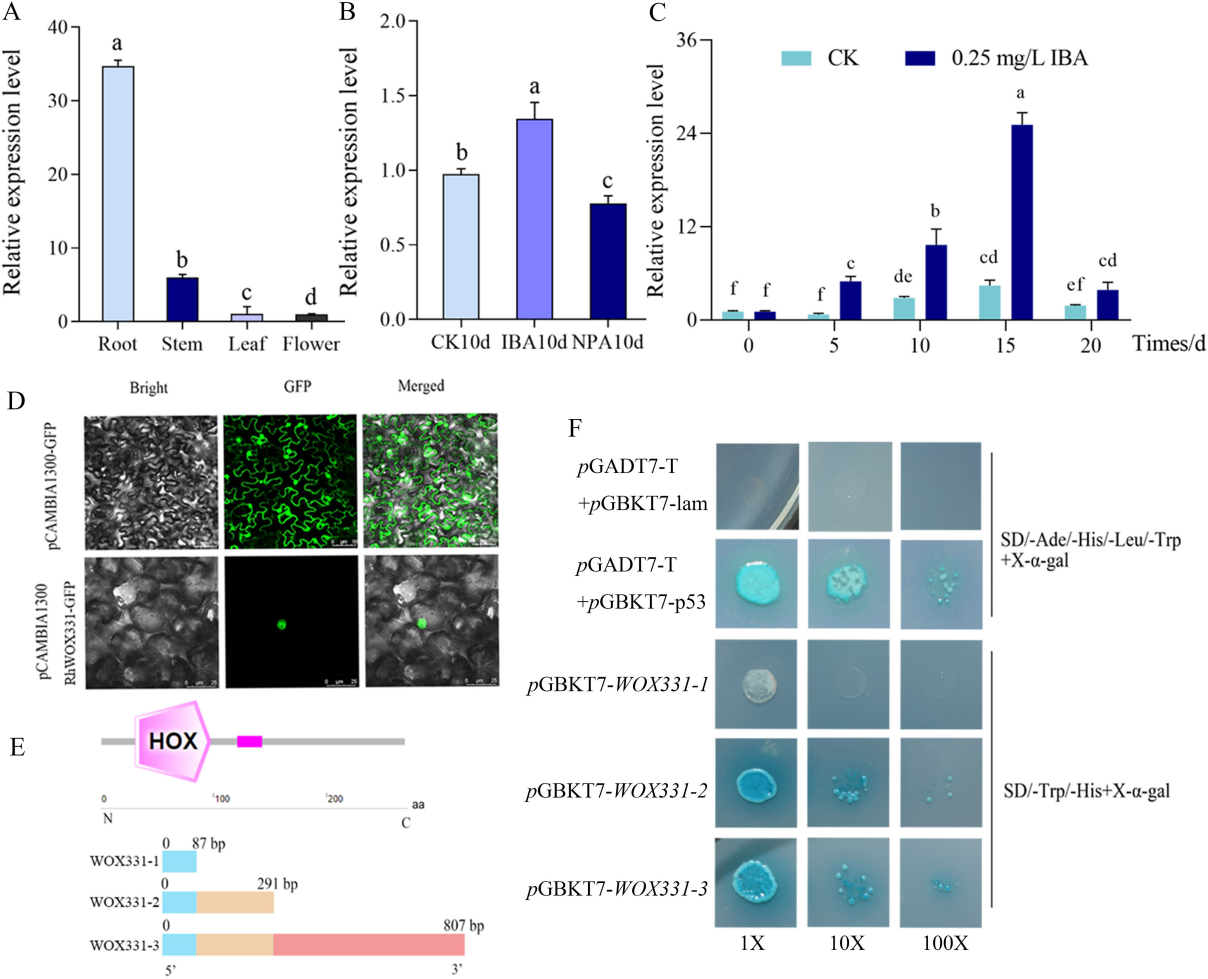
Characterization of *RhWOX331*. **(A)** Expression of *RhWOX331* in different tissues of *R*. ‘The Fairy’ **(B)** Relative expression of *RhWOX331* gene in 0.25 mg/L IBA, 0.2 mg/L NPA-treated and untreated rose cuttings. **(C)** Trend of *RhWOX331* gene expression during adventitious root primordia formation in *R*. ‘The Fairy’ cuttings. **(D)** Subcellular localization of RhWOX331. pCAMBIA1300-GFP empty vector as a control. **(E)** Schematic diagram of yeast self-activation vector construction for *RhWOX331.* **(F)** Verification of yeast self-activation of *RhWOX331*. *p*GADT7-T+*p*GBKT7-p53 served as the positive control, while *p*GADT7-T+*p*GBKT7-lam was utilized as the negative control. Different lowercase letters indicate significant differences among different treatments.

The expression of *RhWOX331* showed tissue-specificity, with the highest expression in roots, followed by stems, and the lowest expression in flowers (**Figure 5A**). Exogenous application of IBA promotes adventitious root formation in roses, whereas NPA application suppresses it. By the 10th day of cutting, exogenous IBA significantly increased the expression of *RhWOX331* to 1.3 times that of the hormone-free control, while exogenous NPA significantly reduced *RhWOX331* expression to 0.8 times that of the hormone-free control (**Figure 5B**). IBA promoted the expression of *RhWOX331* continuously. In the absence of hormones, expression of *RhWOX331* in cuttings remained almost unchanged after 5 d of cultivation. The gene was up-regulated from 5 to 10 days and then down-regulated from 15 to 20 days. After the application of exogenous IBA, *RhWOX331* showed upregulated expression as early as 5 d after culture initiation. At each time point thereafter, the expression level of this gene was significantly higher compared to the control without any hormone addition (**Figure 5C**).

Subcellular localization analysis revealed that in the control tobacco leaf cells, green fluorescence can be observed simultaneously in both the cell membrane and nucleus. In the RhWOX331 group, only green fluorescence was observed within the nucleus, confirming the nuclear localization of RhWOX331 (**Figure 5D**). To verify the transcriptional activation activity of *WOX331*, three segments of the *WOX331* gene were constructed into the *p*GBKT7 vector (**Figure 5E**). On SD/-Ade/-His/-Leu/-Trp medium, the negative control yeast did not grow, while the positive control yeast grew and turned blue after adding X-α-gal. The yeast that transformed *p*GBKT7-*WOX331-1* did not grow, while the yeast that transformed *p*GBKT7-*WOX331-2* and *p*GBKT7-*WOX331-3*, which both containing the HOX domain grew normally and turned blue after adding X-α-gal (**Figure 5F**). This indicates that the transcription factor *RhWOX331* possesses self-activation activity, which may be attributed to the HOX domain spanning amino acids 87 to 807.

### 3.5 The effect of overexpression of *RhWOX331* on rooting and growth of *A. thaliana* seeds

**Figure 6:**
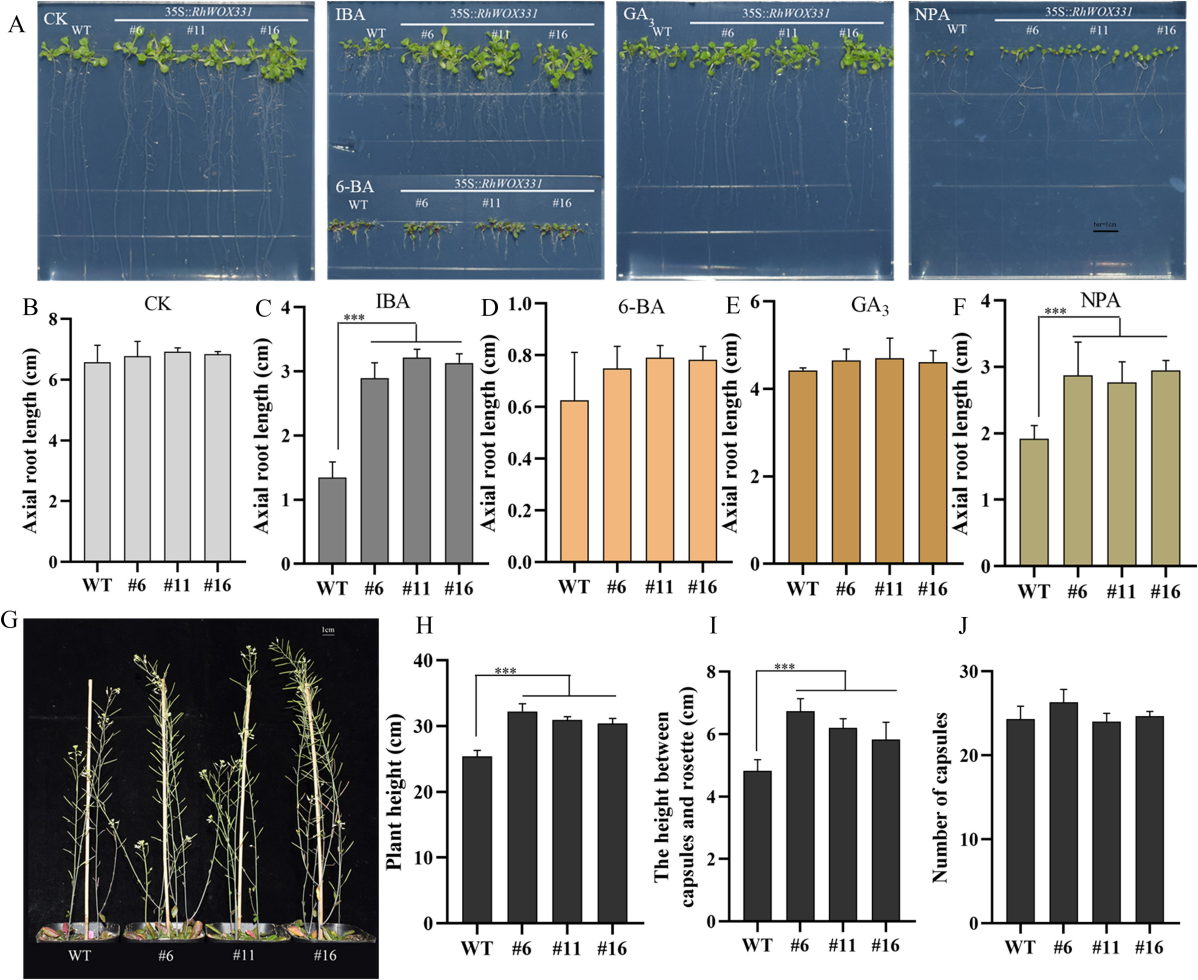
Growth of *A thaliana* seeds overexpressing *RhWOX331*. **(A)** Primary root length of WT and *RhWOX331* overexpressing *A. thaliana* seeds after 14 days of cultivation in different mediums. CK) hormone-free medium; IBA) medium containing 0.25 mg/L IBA; 6-BA) medium containing 0.5 mg/L 6-BA; GA_3_) medium containing 1 mg/L GA_3_; NPA) medium containing 10 μM NPA. **(B-F)** Primary root length of WT and *RhWOX331* overexpressing *A. thaliana* seeds in different medium. Bar = 1 cm. **(B)** hormone-free medium; **(C)** medium containing 0.25 mg/L IBA; **(D)** medium containing 0.5 mg/L 6-BA; **(E)** medium containing 1 mg/L GA_3_; **(F)** medium containing 10 μM NPA. **(G)** The phenotypes of mature WT and *RhWOX331* overexpressing *A. thaliana* plants. Bar = 1 cm. **(H)** The plant height of mature WT and *RhWOX331* overexpressing *A. thaliana* plants. **(I)** The height between the capsules and the rosette of mature WT and *RhWOX331* overexpressing *A. thaliana* plants. **(J)** the number of capsules of mature WT and *RhWOX331* overexpressing *A. thaliana* plants. The *** mark indicates significant difference between WT and transgenic lines.

*RhWOX331*-overexpressing *A. thaliana* lines were obtained and identified to investigate the influence of *RhWOX331* on root development (**Supplementary Figure S3**). There was no significant difference in root length between wild-type (WT) and *RhWOX331*-overexpressing *A. thaliana* plants on hormone-free medium or medium containing 0.5 mg/L 6-BA or 1 mg/L GA_3_ (**Figure 6B**). The average root length of 14-day-old plants was approximately 6.78 cm in the CK, 0.74 cm in the 6-BA group and 4.6cm in the GA_3_ group. Interestingly, on medium containing 0.25 mg/L IBA and 10 μM NPA, *A. thaliana* growth was inhibited, showing differences in root length between WT and transgenic plants (**Figures 6A, C, F**). The root lengths of WT plants were 1.34 cm and 1.91 cm, respectively. However, overexpression of *RhWOX331* alleviated the inhibitory effects of these high concentrations of exogenous hormones, resulting in primary root lengths of 3.08 cm and 2.86 cm, respectively. The number of lateral roots on the primary root had also significantly increased. Moreover, it was found that both the plant height and the height between the capsules and the rosette were increased after overexpressing *RhWOX331* (**Figures 6G, H, I**). It was different that the number of capsules did not increase (**Figure 6J**). These results indicate that overexpression of *RhWOX331* did not promote elongation of primary roots in *A. thaliana*, but enhanced lateral root formation. It also alleviated the inhibitory effects of high concentrations of auxin and auxin inhibitors on primary root elongation. Moreover, *RhWOX331* increased plant height by raising the height between the capsules and the rosettes rather than increasing the number of flowers.

### 3.6 The effect of overexpression of *RhWOX331* on the rooting of *A. thaliana* adventitious roots

**Figure 7:**
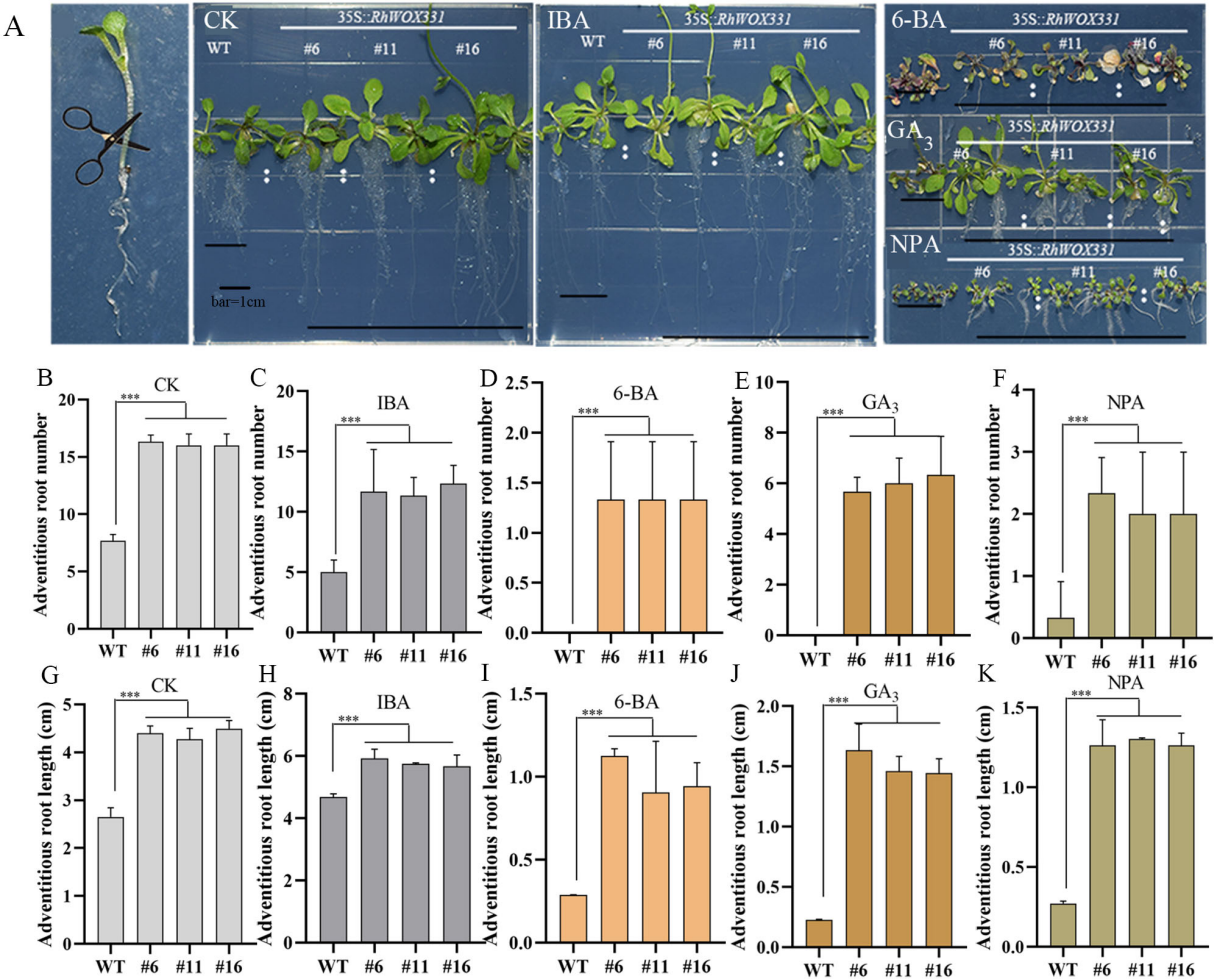
Growth of adventitious roots of WT and *RhWOX331* overexpressing *A. thaliana* in medium containing different hormones. **(A)** After removal of the primary root, WT and *RhWOX331* overexpressing *A. thaliana* developed adventitious roots on medium containing different hormones: CK) hormone-free medium; IBA) medium containing 0.25 mg/L IBA; 6-BA) medium containing 0.5 mg/L 6-BA; GA_3_) medium containing 1 mg/L GA_3_; NPA) medium containing 10 μM NPA. **(B-F)** The number of adventitious roots occurring in WT and overexpressed *RhWOX331 A thaliana* in different medium. Bar = 1 cm. **(B)** hormone-free medium; **(C)** medium containing 0.25 mg/L IBA; **(D)** medium containing 0.5 mg/L 6-BA; **(E)** medium containing 1 mg/L GA_3_; **(F)** medium containing 10 μM NPA. **(G-K)** Length of adventitious roots occurring in WT and overexpressed *RhWOX331 A. thaliana* in different mediums. **(G)** hormone-free medium; **(H)** medium containing 0.25 mg/L IBA; **(I)** medium containing 0.5 mg/L 6-BA; **(J)** medium containing 1 mg/L GA_3_; **(K)** medium containing 10 μM NPA. The *** mark indicates significant difference between WT and transgenic lines.

The primary roots of 14-day-old *A. thaliana* were removed and cultivated on B5 medium containing different hormones. Adventitious root formation in *A. thaliana* was enhanced on hormone-free medium and medium containing 0.25 mg/L IBA. Overexpression lines exhibited earlier adventitious root emergence, with a greater number and longer lengths of adventitious roots compared to the WT (**Figures 7A–C, G, H**). The difference in the number of adventitious roots was particularly pronounced. On the medium containing 0.5 mg/L 6-BA, 1 mg/L GA_3_, and 10 μM NPA, WT plants almost did not form roots after 10 days of culture, whereas *RhWOX331* overexpressing plants developed some adventitious roots (**Figures 7A, D–F, I–K**). In terms of both the number and length of adventitious roots, *RhWOX331* overexpressing *A. thaliana* demonstrated a stronger rooting ability. These results suggest that overexpression of *RhWOX331* promotes adventitious root formation in *A. thaliana* and alleviates the inhibitory effects of some hormones on adventitious root development.

### 3.7 Analysis of GUS activity of *RhWOX331* promoter

**Figure 8:**
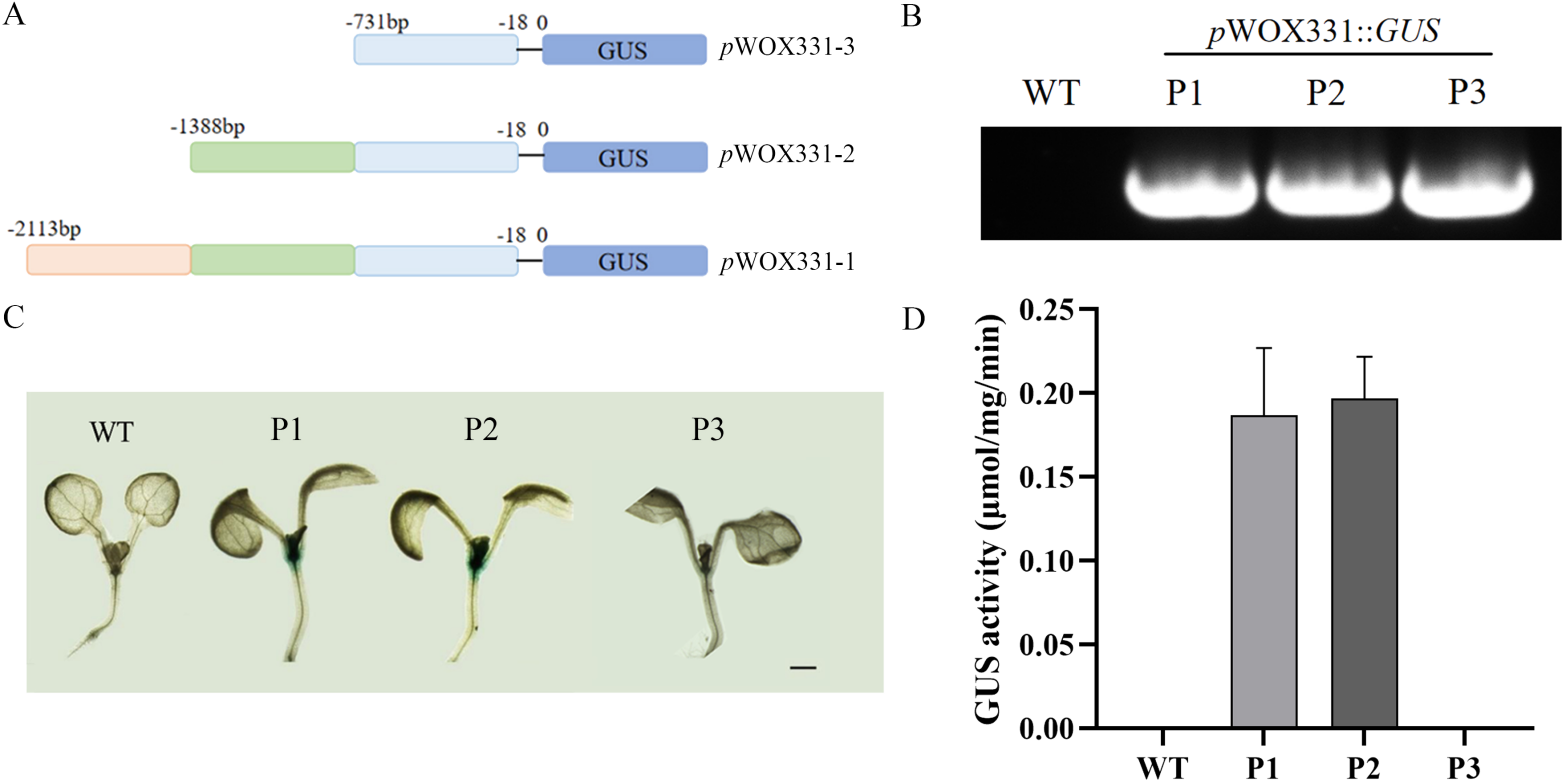
Analysis of GUS activity of *RhWOX331.* **(A)** Schematic diagram of *RhWOX331* GUS vector construction. **(B)** Identification of *A. thaliana* transformed with promoter of *RhWOX331*. P1, P2, and P3 represent *A. thaliana* transformed with *p*WOX331-1::*GUS*, *p*WOX331-2::*GUS* and *p*WOX331-3::*GUS*, respectively. **(C)** GUS staining of WT and *A. thaliana* overexpressing *p*WOX331. Bar = 1 mm. **(D)** GUS activity of WT and *A. thaliana* overexpressing *p*WOX331.

By predicting the approximately 2000bp sequence upstream of the *RhWOX331* gene start codon, it was found that this sequence contained abundant *cis*-regulatory elements (**Supplementary Figure S2**), which may be one of the reasons that *RhWOX331* was regulated by many hormones, such as IBA. According to the position of the TATA-box, the 2113bp sequence was divided into three segments (**Figure 8A**), *p*WOX331-1::*GUS*, *p*WOX331-2::*GUS*, and *p*WOX331-3::*GUS* vectors were constructed and transformed into *A. thaliana* (**Figure 8B**). P1, P2, and P3 represent *A. thaliana* transformed with *p*WOX331-1:: *GUS*, *p*WOX331-2:: *GUS*, and *p*WOX331-3:: *GUS*, respectively. Observation of GUS staining in 7-day-old *A. thaliana* seedlings revealed no blue spots in the WT plants, while GUS signals were detected at the shoot apical meristem and cotyledonary node in overexpressing plants carrying *p*WOX331-1::*GUS* and *p*WOX331-2::*GUS* vectors (**Figure 8C**). GUS activity of *RhWOX331* promoter showed the same results (**Figure 8D**). Considering that no GUS signal was detected in transgenic *A. thaliana* after adventitious root formation, it was speculated that *WOX331* played a role before visible adventitious root formation. These results indicate that the promoter of *WOX331* is located between 731bp and 2113bp. In addition to regulating adventitious root formation, *RhWOX331* also plays a role in the growth point of *A. thaliana* cotyledons.

## 4 Discussion

### 4.1 The *RcWOX* gene family had undergone significant expansion in *Rosa chinensis*

A total of 381 *WOX* genes were identified in rose, a number significantly higher than that found in other species, including 18 in *A. thaliana*, 28 in *N. tabacum*, 26 in *P. trichocarpa* (**Figure 1**), 18 in *Eriobotrya japonica* (Yu et al., 2022) and 33 in *Glycine max* (Hao et al., 2019). The occurrence of more than 100 members in the *WOX* gene family was not unique to roses. Other species within the *Rosa* genus which had published genomes also had a relatively large number of *WOX* genes. *Rosa multiflora* contained 170 *WOX* genes, and 105 *WOX* genes were identified in *Rosa rugosa*. The number of *WOX* genes in rose was substantially higher than that in other species, but in other Rosaceae species, the number of *WOX* genes was not particularly high. There were 9-14 *WOX* gene family members in *Pyrus bretschneideri* and other Rosaceae species (Cao et al., 2017). Lv identified *WOX* gene family members in nine *Prunus* species, ranging from 6 to 40 (Lv et al., 2023). The number of *WOX* genes in *R. chinensis*, *R. multiflora* and *R. rugosa* was also above average, suggesting that the large-scale expansion of the *WOX* gene family was a phenomenon specific to the genus *Rosa*. The genetic background of *R. chinesis* was relatively complex, and *Rosa multiflora* and *Rosa rugosa* might be involved in the breeding process of this species (Cui et al., 2022). The *WOX* gene may have replicated during this process. At present, there is no analysis on the *WOX* function of roses, and more genetic functional evidence is needed to determine the specific significance of this replication process. Whole genome duplications (WGD) are the primary driver of *WOX* family evolution (Cao et al., 2017). In *Bromeliaceae* plants, the CAM-related gene families had experienced accelerated expansion, supporting gene family evolution as a driver of CAM evolution (Groot Crego et al., 2024). Abubakar identified four segmental duplications and one tandem duplication of *WOX* gene family in *Boehmeria nivea* (Abubakar et al., 2023), which suggested that whole-genome duplication (WGD) had contributed to the expansion of the *WOX* gene family in *B. nivea*. During the Paleocene-Eocene boundary, Rosaceae underwent a WGD event, leading to extensive gene duplication (Xiang et al., 2017). The entire *Malus* genus experienced a WGD event, resulting in the duplication of several MADS-box genes potentially linked to pome formation during that period (Zhang et al., 2023). We hypothesize that roses might have undergone WGD during long-term evolution, leading to the expansion of the *RcWOX* gene family, enabling them to adapt to various complex growth environments. After analyzing the collinearity within the rose genome, it is found that the number of collinear genes in rose is 0. Therefore, further data evidence is needed to explain the significant expansion of the *WOX* gene family in rose.

### 4.2 Most of the *WOX* genes had no function during adventitious rooting of rose cuttings

The rooting process of roses is jointly regulated by many genes, but not all members of the *WOX* gene family are involved in this process. Apart from classical clades, most of the *WOX* genes in clades I-V showed no expression during the rose rooting process. Among the 364 *WOX* members from clades I to V, 359 members showed almost no expression during the rooting process of rose, with only *RhWOX276*, *RhWOX51*, *RhWOX33*, *RhWOX284*, and *RhWOX372* genes exhibiting transcriptional expression counts higher than 10 during three or more periods. The classical clades in *RcWOX* gene family members in rose demonstrated similar structures and the presence of UTR in most cases. Conversely, the majority of *RcWOX* family members in clades I-V exhibited UTR loss (**Supplementary Figure S1**). Similar to classical clades in rose, 14 out of 16 pairs of homologous genes in the soybean *GmWOX* gene family exhibited relatively conserved exon/intron structures (Hao et al., 2019). Many genes in *WOX* gene family of rose did not function during the formation of adventitious roots, while the genes in classical clades exhibited relatively high expression levels, suggesting that these genes might play a role in the rose cutting rooting process. Multiple *WOX* gene family members in different stages of rose rooting responded to cutting signals, such as *RcaWOX1* in *R. canina* callus tissue formation at an early stage (Gao et al., 2014), similar to the expression pattern of *RhWOX185* in *R*. ‘The Fairy’. The homologous gene *MdWOX11* of *RhWOX331* in apple cuttings reached its highest expression level at 3 days, and its expression was inhibited by 6-BA (Mao et al., 2023), corresponding with the expression trends of genes *RhWOX372*, *RhWOX316*, and *RhWOX271* in rose. In conclusion, the *WOX* gene of clades I-V regulating the functions of other aspects of roses require further investigation.

### 4.3 *RhWOX331* in *R. hybrida* can regulate plant meristem activity

Further research on the expression pattern and function of *RhWOX331* in plants revealed that it not only played a role in adventitious root development, but may also be related to plant meristem activity and regulated the development of aboveground and underground parts of plants. Compared to other tissues, the expression level of *RhWOX331* gene in rose roots was significantly increased (**Figure 5A**), similarly, *WOX* genes in poplar were primarily expressed in roots and leaves (Liu et al., 2014). In *Triticum aestivum*, the homologous gene *TaWOX11* of *RhWOX331* was also highly expressed in roots compared to other tissues. In addition, both *TaWUS* and *TaWOX9* were transcriptional activators and the transcription activation regions were located at the C-terminus (Li et al., 2020).

Following IBA signaling, the expression of *RhWOX331* was upregulated and its functional role was advanced during the rooting process (**Figure 5C**). Overexpression of *RhWOX331* in *A. thaliana* demonstrated enhanced primary root and adventitious root formation, indicating the role of *RhWOX331* in promoting primary root elongation and adventitious root development in plants (**Figures 6**, **7**). Similarly, in *A. thaliana*, *AtWOX11* and *AtWOX12* responded to auxin signals, inducing fate transition of stem cells from the pericycle cells to root founder cells, thereby inducing adventitious root formation (Liu and Xu, 2018). *AtWOX11* was involved in the transition of vascular cambium cells to new lateral root primordia primordial cells (Baesso et al., 2018).

The *RhWOX331* promoter, *p*WOX331-1 and *p*WOX331-2, triggers GUS protein expression in the meristematic region, indicating the gene’s regulation of plant meristematic activity (**Figure 8**). Additionally, auxin signaling can be detected in this area during *A. thaliana* embryogenesis (Baesso et al., 2018), suggesting that *p*WOX331-2 may overlap with auxin signaling to regulate embryonic development. Indeed, during adventitious root formation in *A. thaliana*, the distribution of auxin response coincides with the expression region of *WOX11*, directly responding to the maximum auxin level in the wound-induced pericycle. In rice crown root development, *WOX11* might integrate auxin and cytokinin signaling to regulate the expression of RR2 (Type-A cytokinin-responsive regulator) genes in the crown root primordium, thereby regulating cell proliferation (Zhao et al., 2009). *WOX* gene family played an important role in embryogenesis and shoot apical meristem establishment in conifers (Bueno et al., 2021). Therefore, we propose that *RhWOX331* can respond to auxin signals, regulate plant meristematic activity, and positively correlate with the development of both aboveground and underground parts of plants.

## 5 Conclusions

The study identified 381 *WOX* genes in *Rosa chinensis* through whole-genome bioinformatics analysis. Phylogenetic analysis and evolutionary tree construction classified the *RcWOX* gene family into eight clades. Gene structure and promoter *cis*-element analysis revealed that genes within the same clade exhibit similar structures and functions. Chromosomal localization of *RcWOX* genes in roses indicated significant expansion on chromosome 2. Relative expression analysis of nine *WOX* gene family members during rose rooting identified several genes with significant expression changes in this process. The *RhWOX331* gene, potentially associated with rooting, was identified through tissue-specific expression analysis, showing high expression in roots and inducibility by IBA while being suppressed by NPA. *RhWOX331* located to the nucleus and exhibited yeast self-activation activity. Overexpression of the *RhWOX331* gene significantly increased the number of lateral roots on the primary root and enhanced the height of *A. thaliana*. Additionally, it accelerated adventitious root formation and alleviated the inhibition of adventitious root initiation by certain hormones. This gene functioned at the growth point of *A. thaliana* cotyledons. Our study provides initial insights into the role of *RhWOX331* in the process of adventitious root formation in *R.* ‘The Fairy’, offering direction and inspiration for future research on the *WOX* gene family of rose.

## Data Availability

The original contributions presented in the study are included in the article/**Supplementary Material**, further inquiries can be directed to the corresponding author/s.

## Supplementary material

Supplementary Figure 1 Conserved motifs and conserved domains of *RcWOXs* in *Rosa chinensis.* **(A)** Motif composition of RcWOX proteins, with different colors representing twenty distinct motifs. **(B)** Conserved domains of RcWOXs, with various colors indicating different structural domains. **(C)** Green rectangles denote untranslated regions (UTRs); yellow rectangles represent coding sequences (CDS) or exons; black lines indicate introns.

Supplementary Figure 2
*Cis*-acting element analysis of *RcWOXs*. Each *cis*-acting element is indicated by a different color.

Supplementary Figure 3 Characterization of *A. thaliana* overexpressing *RhWOX331*.
