# Supplementary figures and images for: Genome-wide analysis of the WOX gene family and function exploration of RhWOX331 in rose (R. ‘The Fairy’)
Source: Front Plant Sci. 2024 Sep 3;15:1461322. doi: 10.3389/fpls.2024.1461322 (PMC11405225; doi:10.3389/fpls.2024.1461322)

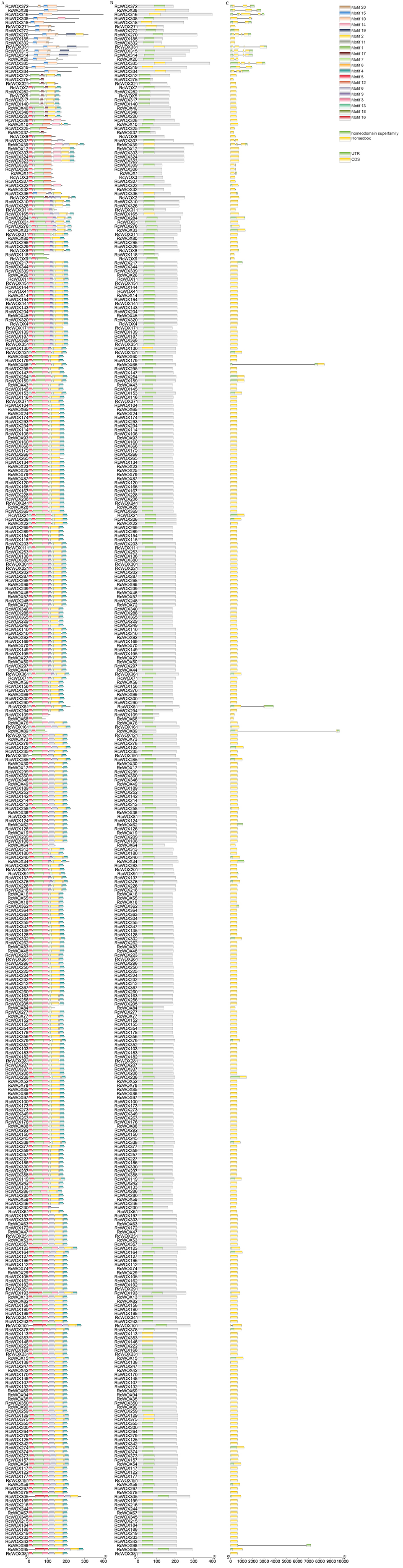

Supplement: Supplementary Figure 1 — Conserved motifs and conserved domains of RcWOXs in Rosa chinensis. (A) Motif composition of RcWOX proteins, with different colors representing twenty distinct motifs. (B) Conserved domains of RcWOXs, with various colors indicating different structural domains. (C) Green rectangles denote untranslated regions (UTRs); yellow rectangles represent coding sequences (CDS) or exons; black lines indicate introns. [file Image1.jpg]

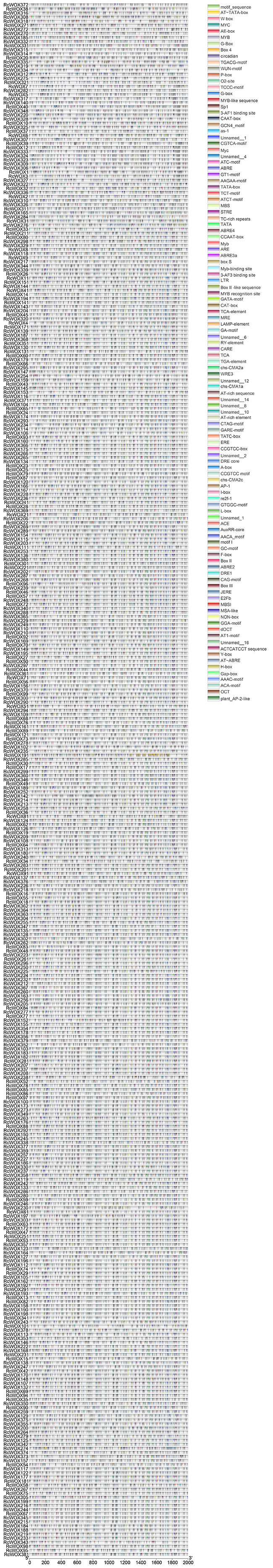

Supplement: Supplementary Figure 2 — Cis-acting element analysis of RcWOXs. Each cis-acting element is indicated by a different color. [file Image2.jpg]

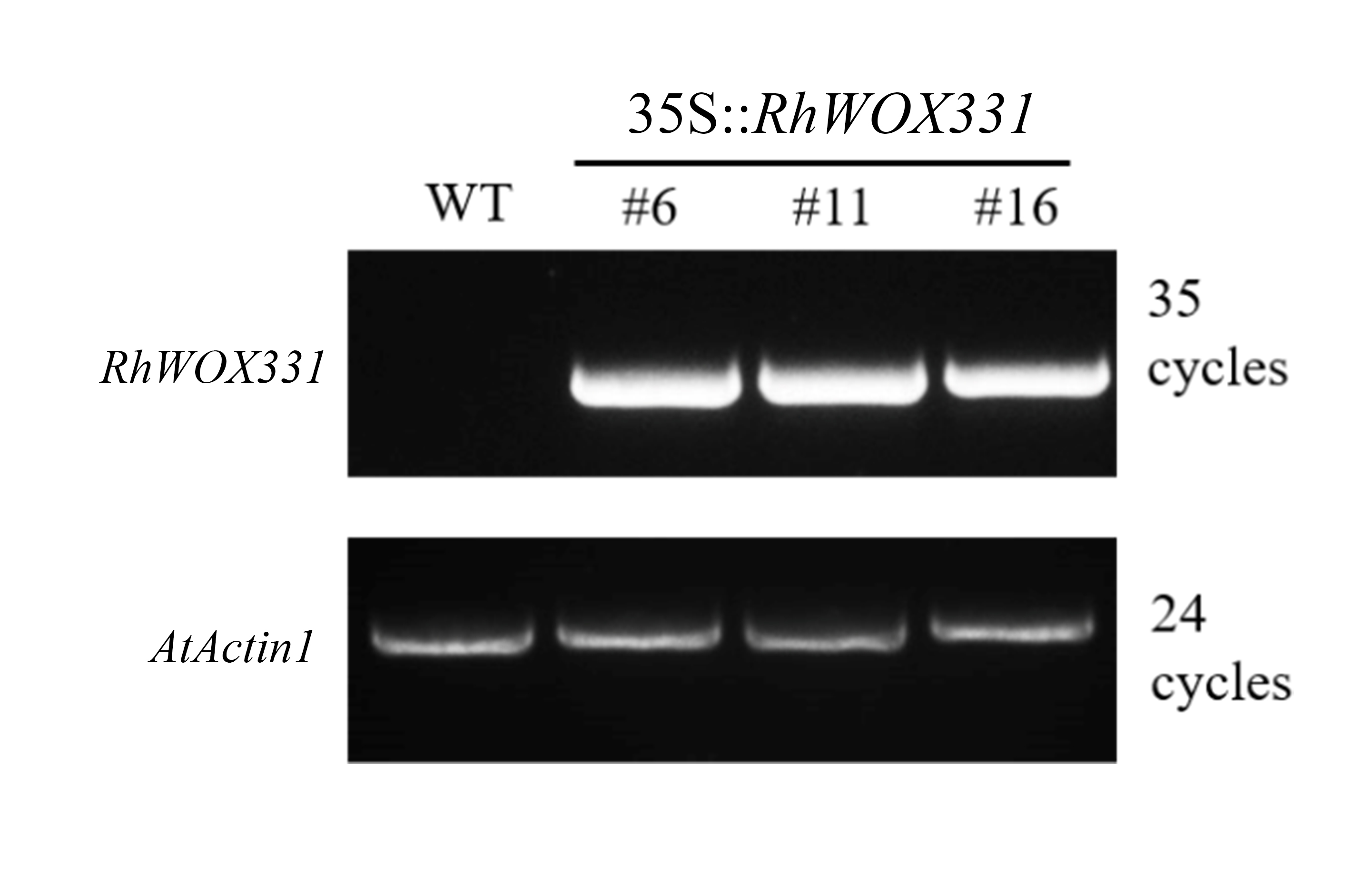

Supplement: Supplementary Figure 3 — Characterization of A. thaliana overexpressing RhWOX331. [file Image3.jpeg]
