# Supplementary material for: Genome-wide analysis of the WOX gene family and function exploration of RhWOX331 in rose (R. ‘The Fairy’)
Source: Front Plant Sci. 2024 Sep 3;15:1461322. doi: 10.3389/fpls.2024.1461322 (PMC11405225; doi:10.3389/fpls.2024.1461322)
Supplement: Supplementary file 5 [file Table2.docx]

| Primer name | Primer sequence |
| --- | --- |
| RhWOX284qF | TGTTCCCATGCAAAGATCAGG |
| RhWOX284qR | ATCCATAACCGCCATAGTTCC |
| RhWOX372qF | TGATGGGTTACAACGATGGTG |
| RhWOX372qR | AGGTGGAGCATGTGTACTTG |
| RhWOX316qF | AGAAGTCGGTGCTGAACATG |
| RhWOX316qR | GGGAAGAGTTGGAGTGTTTTAAG |
| RhWOX308qF | AAGACAAAAGCAGAAACGCAG |
| RhWOX308qR | CCCAAACAATCAAATGCCCAG |
| RhWOX318qF | AGGAGCTTTACGATGACAAGG |
| RhWOX318qR | AGCCCTTTGATTCTGGAACC |
| RhWOX271qF | GAGGGTTGAAGGCAAGAATGTA |
| RhWOX271qR | CTTTGTCGTGGAGACTGCTAAT |
| RhWOX270qF | AGTCCCAACAGAACACATCAG |
| RhWOX270qR | GACATATGGCCTAAACCTCTCG |
| RhWOX185qF | ATGGCTTCATCAAACAGACACT |
| RhWOX185qR | GCACGATAGAGGGATTGATTAC |
| RhWOX331qF | GCAAACGTCTTTTACTGGTTCC |
| RhWOX331qR | GCTTCCATACTGAATTGCACC |
| WOX331F | ATGGGAATGAGCAGCATGAAG |
| WOX331R | TCATCTCATGCCTTCTGGGC |
| RhActinF | TGAGGCCATTTACGACAT |
| RhActinR | AGATCACAGGAGCATAGGAG |
| AtActin1F | GAAAATGGCTGATGGTGAAG |
| AtActin1R | CTCATAGATAGGAACAGTGTGGC |
| CEWOX331P1F | gaccatgattacgccaagcttGCTTAGAAAACTTGGTAGCAGCTAG |
| Primer name | Primer sequence |
| CEWOX331P2F | gaccatgattacgccaagcttATCTTTTTTGGATTTGACTTGAACTT |
| CEWOX331P3F | gaccatgattacgccaagcttATTGGGGGACGATATACATTTTATT |
| CEWOX331R | ggactgaccacccggggatccTACTGTGAGGAAGGAAGGAGAGAAG |
| GUSF | ATGTTACGTCCTGTAGAAACCCCA |
| GUSR | TCATTGTTTGCCTCCCTGCTG |
